# Supplementary material for: Does the Type Matter? Verification of Different Tea Types’ Potential in the Synthesis of SeNPs
Source: Antioxidants (Basel). 2022 Dec 18;11(12):2489. doi: 10.3390/antiox11122489 (PMC9774132; doi:10.3390/antiox11122489)
Supplement: Supplementary file 1 [file antioxidants-11-02489-s001.zip › antioxidants-2087040-supplementary.pdf]

Supplementary material

Does the Type Matter? Verification of Different Tea Types' Potential in the Synthesis of SeNPs

Aleksandra Sentkowska, Krystyna Pyrzynska

Table S1. The concentrations of polyphenolic compounds in postreaction mixture (in mg L^{-1}).

	Postreaction mixture			
	BSeNPs	GSeNPs	RSeNPs	WSeNPs
EGCG	0.659	0.263	0.058	0.133
Protocatechuic acid	0.152	0.050	0.156	0.185
Epicatechin	0.155	0.111	0.175	0.103
Catechin	<LOD	<LOD	<LOD	<LOD
p-cumaric acid	4.750	1.670	2.186	5.03
Chlorogenic acid	<LOD	<LOD	<LOD	<LOD
pHBA	19.70	2.236	9.545	7.865
Rutin	<LOD	<LOD	<LOD	<LOD
Caffeic acid	<LOD	<LOD	<LOD	<LOD
Gallic acid	0.244	0.168	0.149	0.134

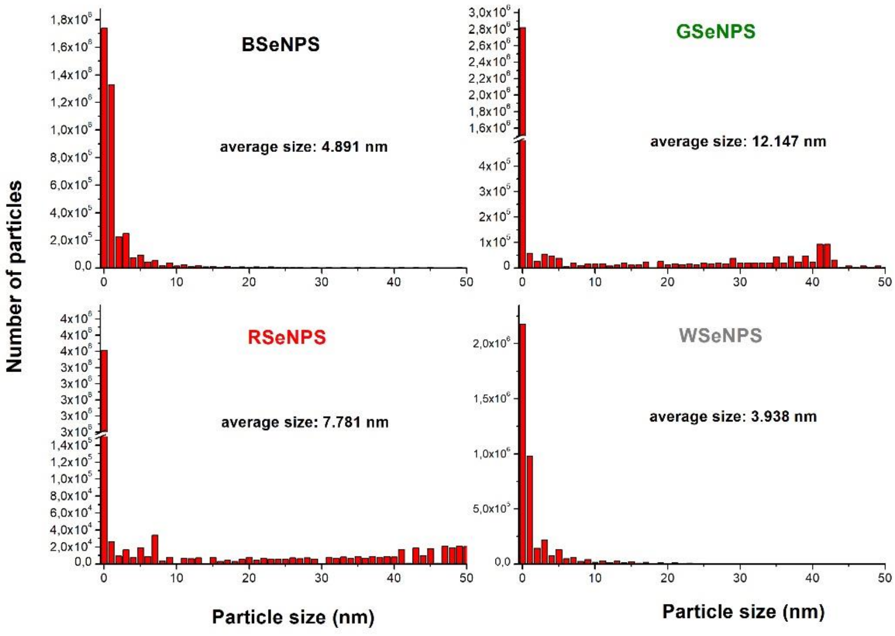

Figure S1. Size distribution of the synthesized SeNPs.

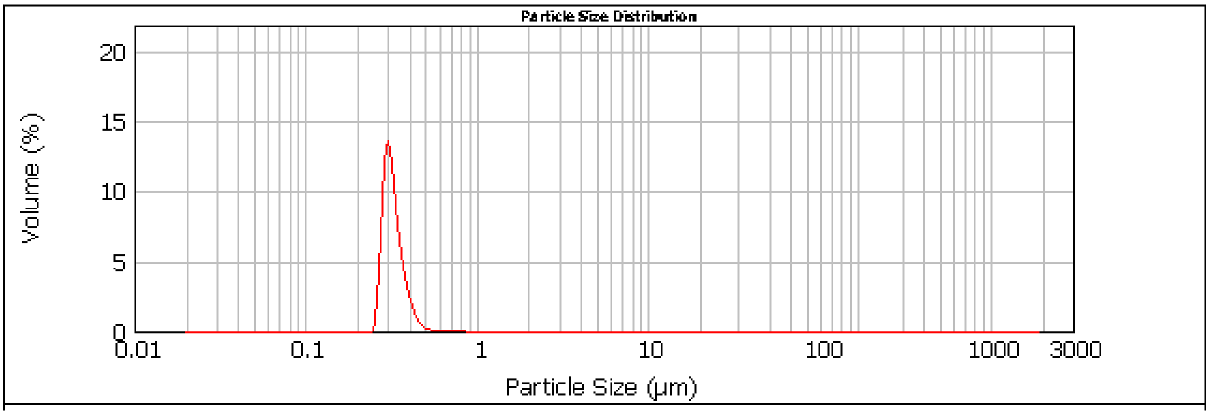

Figure S2. Particle size distribution in GSeNPs obtained by DLS method.

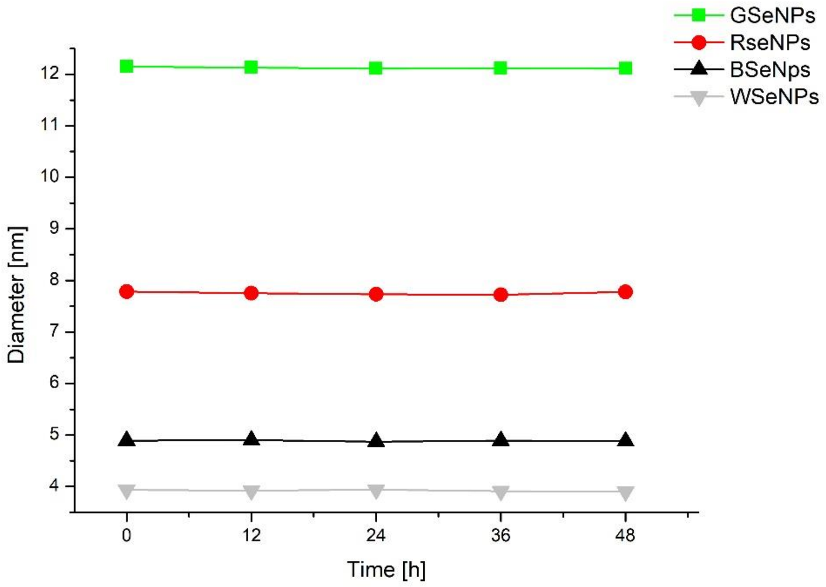

Figure S3. 48h stability of an average diameter of obtained SeNPs.

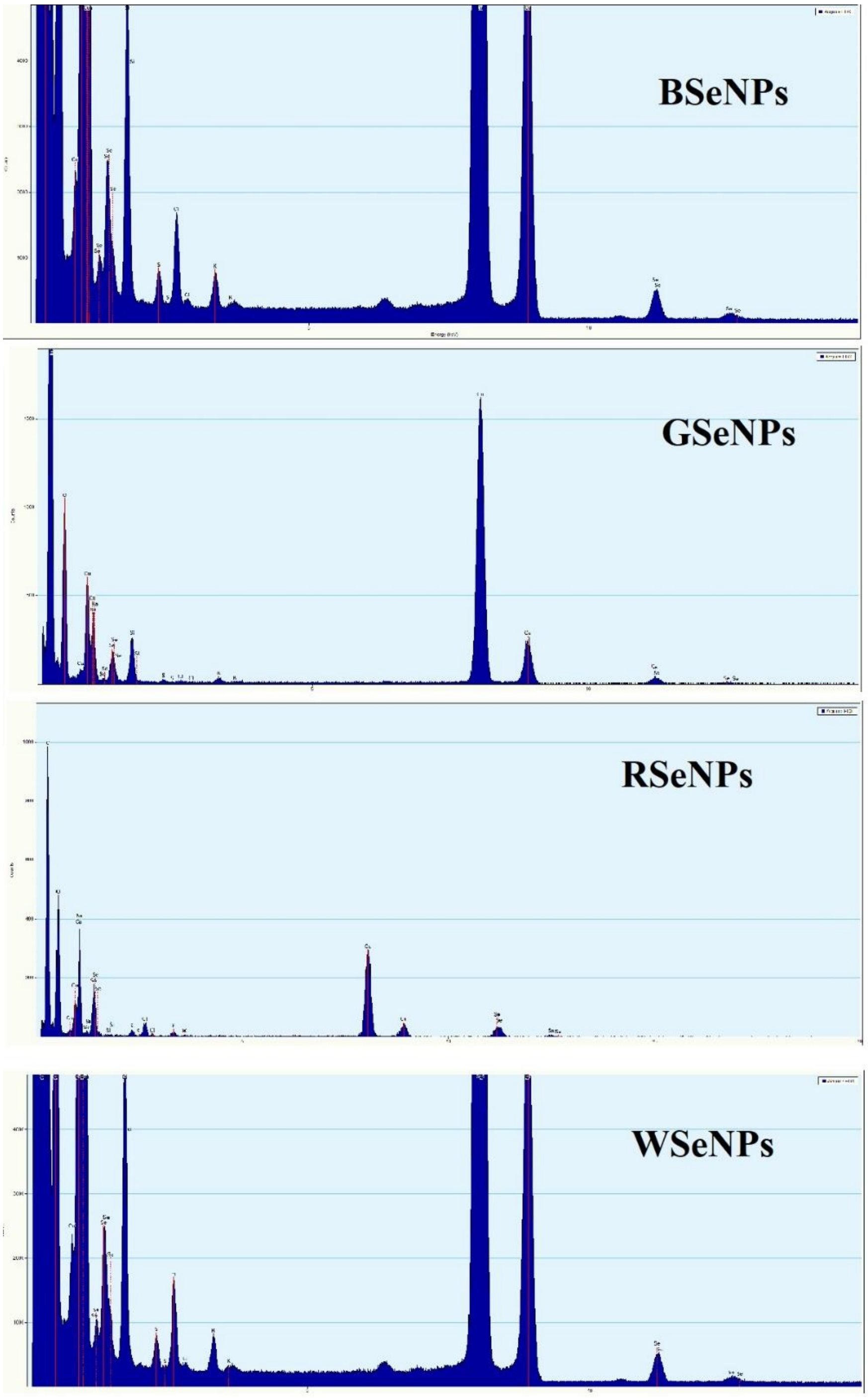

Figure S4. EDS spectra of BSeNPs. GSeNPs, RSeNPs and WSeNPs.
